# Nanomedicine: Principles, Properties, and Regulatory Issues

**DOI:** 10.3389/fchem.2018.00360

**Published:** 2018-08-20

**Authors:** Sara Soares, João Sousa, Alberto Pais, Carla Vitorino

**Affiliations:** ^1^Faculty of Pharmacy, University of Coimbra, Coimbra, Portugal; ^2^REQUIMTE/LAQV, Group of Pharmaceutical Technology, Faculty of Pharmacy, University of Coimbra, Coimbra, Portugal; ^3^Department of Chemistry, Coimbra Chemistry Centre, University of Coimbra, Coimbra, Portugal; ^4^Center for Neurosciences and Cell Biology, University of Coimbra, Coimbra, Portugal

**Keywords:** nanotechnology, nanomedicine, nanomaterials, pharmaceutical development, nanotoxicology

## Abstract

Several scientific areas have benefited significantly from the introduction of nanotechnology and the respective evolution. This is especially noteworthy in the development of new drug substances and products. This review focuses on the introduction of nanomedicines in the pharmaceutical market, and all the controversy associated to basic concepts related to these nanosystems, and the numerous methodologies applied for enhanced knowledge. Due to the properties conferred by the nanoscale, the challenges for nanotechnology implementation, specifically in the pharmaceutical development of new drug products and respective regulatory issues are critically discussed, mainly focused on the European Union context. Finally, issues pertaining to the current applications and future developments are presented.

## Introduction

Over the last years, nanotechnology has been introduced in our daily routine. This revolutionary technology has been applied in multiple fields through an integrated approach. An increasing number of applications and products containing nanomaterials or at least with nano-based claims have become available. This also happens in pharmaceutical research. The use of nanotechnology in the development of new medicines is now part of our research and in the European Union (EU) it has been recognized as a Key Enabling Technology, capable of providing new and innovative medical solution to address unmet medical needs (Bleeker et al., 2013; Ossa, 2014; Tinkle et al., 2014; Pita et al., 2016).

The application of nanotechnology for medical purposes has been termed nanomedicine and is defined as the use of nanomaterials for diagnosis, monitoring, control, prevention and treatment of diseases (Tinkle et al., 2014). However, the definition of nanomaterial has been controversial among the various scientific and international regulatory corporations. Some efforts have been made in order to find a consensual definition due to the fact that nanomaterials possess novel physicochemical properties, different from those of their conventional bulk chemical equivalents, due to their small size. These properties greatly increase a set of opportunities in the drug development; however, some concerns about safety issues have emerged. The physicochemical properties of the nanoformulation which can lead to the alteration of the pharmacokinetics, namely the absorption, distribution, elimination, and metabolism, the potential for more easily cross biological barriers, toxic properties and their persistence in the environment and human body are some examples of the concerns over the application of the nanomaterials (Bleeker et al., 2013; Tinkle et al., 2014).

To avoid any concern, it is necessary establishing an unambiguous definition to identify the presence of nanomaterials. The European Commission (EC) created a definition based on the European Commission Joint Research Center and on the Scientific Committee on Emerging and Newly Identified Health Risks. This definition is only used as a reference to determine whether a material is considered a nanomaterial or not; however, it is not classified as hazardous or safe. The EC claims that it should be used as a reference for additional regulatory and policy frameworks related to quality, safety, efficacy, and risks assessment (Bleeker et al., 2013; Boverhof et al., 2015).

## Nanomaterial

### Definition

According to the EC recommendation, nanomaterial refers to a natural, incidental, or manufactured material comprising particles, either in an unbound state or as an aggregate wherein one or more external dimensions is in the size range of 1–100 nm for ≥50% of the particles, according to the number size distribution. In cases of environment, health, safety or competitiveness concern, the number size distribution threshold of 50% may be substituted by a threshold between 1 and 50%. Structures with one or more external dimensions below 1 nm, such as fullerenes, graphene flakes, and single wall carbon nanotubes, should be considered as nanomaterials. Materials with surface area by volume in excess of 60 m^2^/cm^3^ are also included (Commission Recommendation., 2011). This defines a nanomaterial in terms of legislation and policy in the European Union. Based on this definition, the regulatory bodies have released their own guidances to support drug product development.

The EMA working group introduces nanomedicines as purposely designed systems for clinical applications, with at least one component at the nanoscale, resulting in reproducible properties and characteristics, related to the specific nanotechnology application and characteristics for the intended use (route of administration, dose), associated with the expected clinical advantages of nano-engineering (e.g., preferential organ/tissue distribution; Ossa, 2014).

Food and Drug Administration (FDA) has not established its own definition for “nanotechnology,” “nanomaterial,” “nanoscale,” or other related terms, instead adopting the meanings commonly employed in relation to the engineering of materials that have at least one dimension in the size range of approximately 1 nanometer (nm) to 100 nm. Based on the current scientific and technical understanding of nanomaterials and their characteristics, FDA advises that evaluations of safety, effectiveness, public health impact, or regulatory status of nanotechnology products should consider any unique properties and behaviors that the application of nanotechnology may impart (Guidance for Industry, FDA, 2014).

According to the former definition, there are three fundamental aspects to identify the presence of a nanomaterial, which are size, particle size distribution (PSD) and surface area (Commission Recommendation., 2011; Bleeker et al., 2013; Boverhof et al., 2015).

#### Size

The most important feature to take into account is size, because it is applicable to a huge range of materials. The conventional range is from 1 to 100 nm. However, there is no bright line to set this limit. The maximum size that a material can have to be considered nanomaterial is an arbitrary value because the psychochemical and biological characteristics of the materials do not change abruptly at 100 nm. To this extent, it is assumed that other properties should be taken in account (Lövestam et al., 2010; Commission Recommendation., 2011; Bleeker et al., 2013; Boverhof et al., 2015).

**Figure 1:**
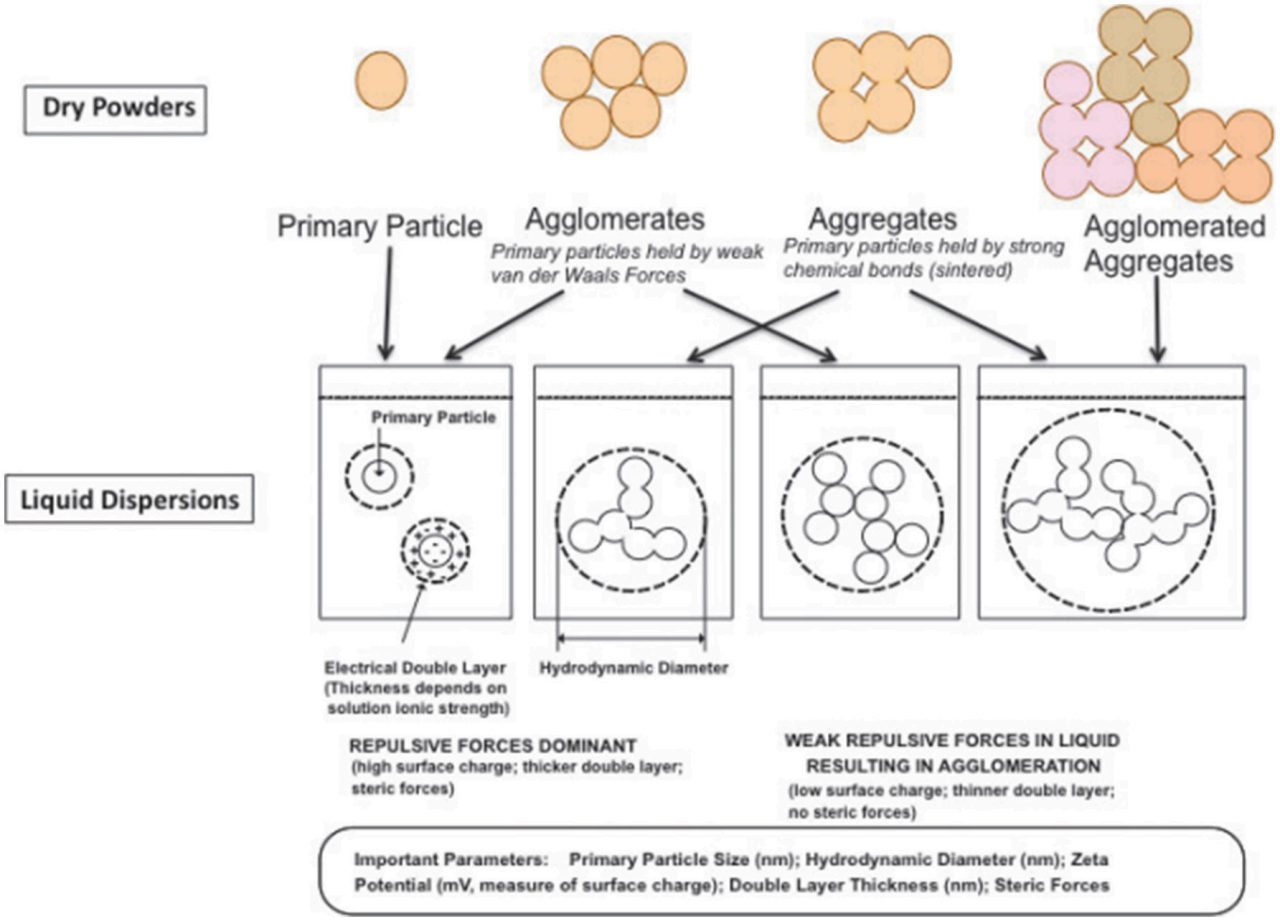
Schematic representation of the different forms of particles: primary particle, aggregate, and agglomerate (reproduced with permission from Oberdörster, 2010).

The pharmaceutical manufacturing of nanomaterials involves two different approaches: top down and bottom down. The top down process involves the breakdown of a bulk material into a smaller one or smaller pieces by mechanical or chemical energy. Conversely, the bottom down process starts with atomic or molecular species allowing the precursor particles to increase in size through chemical reaction (Luther, 2004; Oberdörster, 2010; Boverhof et al., 2015). These two processes of manufacturing are in the origin of different forms of particles termed primary particle, aggregate and agglomerate (Figure 1). The respective definition is *(sic)*:

“particle is a minute piece of matter with defined physical boundaries” (Oberdörster, 2010; Commission Recommendation., 2011);

“aggregate denotes a particle comprising strongly bound or fused particles”—and the external surface can be smaller than the sum of the surface areas of the individual particles (Oberdörster, 2010; Commission Recommendation., 2011);

“agglomerate means a collection of weakly bound particles or aggregates where the resulting external surface area are similar to the sum of the surface areas of the individual components” (Oberdörster, 2010; Commission Recommendation., 2011).

Considering the definition, it is understandable why aggregates and agglomerates are included. They may still preserve the properties of the unbound particles and have the potential to break down in to nanoscale (Lövestam et al., 2010; Boverhof et al., 2015). The lower size limit is used to distinguish atoms and molecules from particles (Lövestam et al., 2010).

#### Particle size distribution

The PSD is a parameter widely used in the nanomaterial identification, reflecting the range of variation of sizes. It is important to set the PSD, because a nanomaterial is usually polydisperse, which means, it is commonly composed by particles with different sizes (Commission Recommendation., 2011; Bleeker et al., 2013; Boverhof et al., 2015).

#### Surface area

The determination of the surface area by volume is a relational parameter, which is necessary when requested by additional legislation. The material is under the definition if the surface area by volume is larger than 60 m^2^/cm^3^, as pointed out. However, the PSD shall prevail, and for example, a material is classified as a nanomaterial based on the particle size distribution, even if the surface area by volume is lower than the specified 60 m^2^/cm^3^ (Commission Recommendation., 2011; Bleeker et al., 2013; Boverhof et al., 2015).

### Dynamic behavior of nanomaterials and applications in nanomedicine

Nanomaterials can be applied in nanomedicine for medical purposes in three different areas: diagnosis (nanodiagnosis), controlled drug delivery (nanotherapy), and regenerative medicine. A new area which combines diagnostics and therapy termed theranostics is emerging and is a promising approach which holds in the same system both the diagnosis/imaging agent and the medicine. Nanomedicine is holding promising changes in clinical practice by the introduction of novel medicines for both diagnosis and treatment, having enabled to address unmet medical needs, by (i) integrating effective molecules that otherwise could not be used because of their high toxicity (e.g., Mepact), (ii) exploiting multiple mechanisms of action (e.g., Nanomag, multifunctional gels), (iii) maximizing efficacy (e.g., by increasing bioavailability) and reducing dose and toxicity, (iv) providing drug targeting, controlled and site specific release, favoring a preferential distribution within the body (e.g., in areas with cancer lesions) and improved transport across biological barriers (Chan, 2006; Méndez-Rojas et al., 2009; Zhang et al., 2012; Ossa, 2014).

This is a result of intrinsic properties of nanomaterials that have brought many advantages in the pharmaceutical development. Due to their small size, nanomaterials have a high specific surface area in relation to the volume. Consequently, the particle surface energy is increased, making the nanomaterials much more reactive. Nanomaterials have a tendency to adsorb biomolecules, e.g., proteins, lipids, among others, when in contact with the biological fluids. One of the most important interactions with the living matter relies on the plasma/serum biomoleculeadsorption layer, known as “corona,” that forms on the surface of colloidal nanoparticles (Pino et al., 2014). Its composition is dependent on the portal of entry into the body and on the particular fluid that the nanoparticles come across with (e.g., blood, lung fluid, gastro-intestinal fluid, etc.). Additional dynamic changes can influence the “corona” constitution as the nanoparticle crosses from one biological compartment to another one (Pearson et al., 2014; Louro, 2018).

Furthermore, optical, electrical and magnetic properties can change and be tunable through electron confinement in nanomaterials. In addition, nanomaterials can be engineered to have different size, shape, chemical composition and surface, making them able to interact with specific biological targets (Oberdörster et al., 2005; Kim et al., 2010). A successful biological outcome can only be obtained resorting to careful particle design. As such, a comprehensive knowledge of how the nanomaterials interact with biological systems are required for two main reasons.

The first one is related to the physiopathological nature of the diseases. The biological processes behind diseases occur at the nanoscale and can rely, for example, on mutated genes, misfolded proteins, infection by virus or bacteria. A better understanding of the molecular processes will provide the rational design on engineered nanomaterials to target the specific site of action desired in the body (Kim et al., 2010; Albanese et al., 2012). The other concern is the interaction between nanomaterial surface and the environment in biological fluids. In this context, characterization of the biomolecules corona is of utmost importance for understanding the mutual interaction nanoparticle-cell affects the biological responses. This interface comprises dynamic mechanisms involving the exchange between nanomaterial surfaces and the surfaces of biological components (proteins, membranes, phospholipids, vesicles, and organelles). This interaction stems from the composition of the nanomaterial and the suspending media. Size, shape, surface area, surface charge and chemistry, energy, roughness, porosity, valence and conductance states, the presence of ligands, or the hydrophobic/ hydrophilic character are some of the material characteristics that influence the respective surface properties. In turn, the presence of water molecules, acids and bases, salts and multivalent ions, surfactants are some of the factors related to the medium that will influence the interaction. All these aspects will govern the characteristics of the interface between the nanomaterial and biological components and, consequently, promote different cellular fates (Nel et al., 2009; Kim et al., 2010; Albanese et al., 2012; Monopoli et al., 2012).

A deeper knowledge about how the physicochemical properties of the biointerface influence the cellular signaling pathway, kinetics and transport will thus provide critical rules to the design of nanomaterials (Nel et al., 2009; Kim et al., 2010; Albanese et al., 2012; Monopoli et al., 2012).

## Challenges in pharmaceutical development

**Figure 2:**
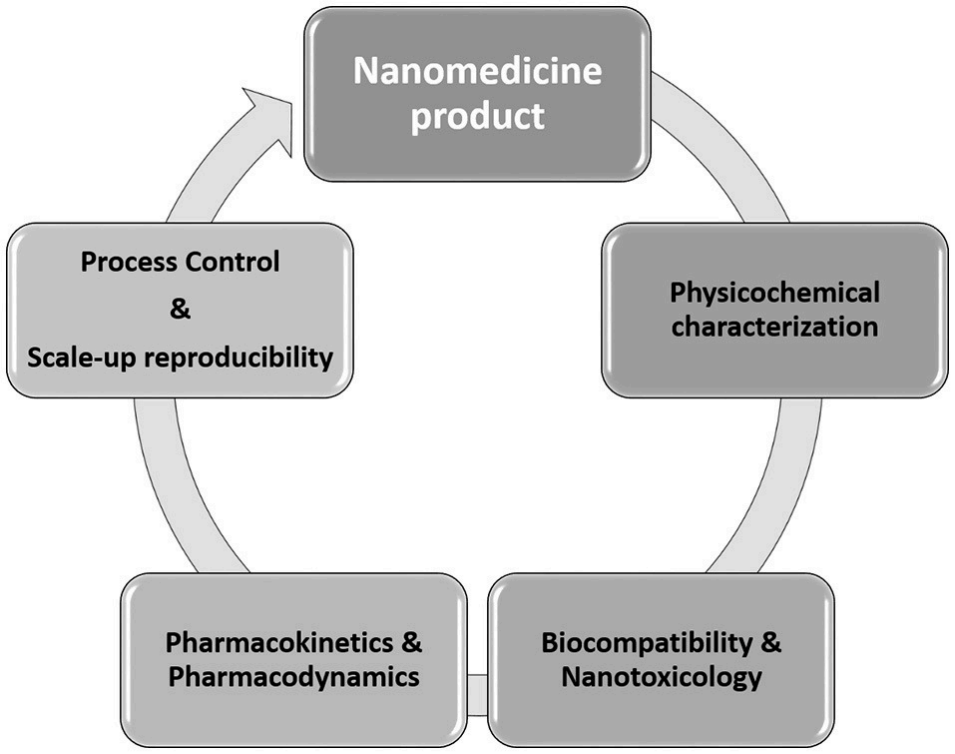
Schematic representation of the several “barriers” found throughout the development of a nanomedicine product.

The translation of nanotechnology form the bench to the market imposed several challenges. General issues to consider during the development of nanomedicine products including physicochemical characterization, biocompatibility, and nanotoxicology evaluation, pharmacokinetics and pharmacodynamics assessment, process control, and scale-reproducibility (Figure 2) are discussed in the sections that follow.

### Physicochemical characterization

The characterization of a nanomedicine is necessary to understand its behavior in the human body, and to provide guidance for the process control and safety assessment. This characterization is not consensual in the number of parameters required for a correct and complete characterization. Internationally standardized methodologies and the use of reference nanomaterials are the key to harmonize all the different opinions about this topic (Lin et al., 2014; Zhao and Chen, 2016).

Ideally, the characterization of a nanomaterial should be carried out at different stages throughout its life cycle, from the design to the evaluation of its *in vitro* and *in vivo* performance. The interaction with the biological system or even the sample preparation or extraction procedures may modify some properties and interfere with some measurements. In addition, the determination of the *in vivo* and *in vitro* physicochemical properties is important for the understanding of the potential risk of nanomaterials (Lin et al., 2014; Zhao and Chen, 2016).

The Organization for Economic Co-operation and Development started a Working Party on Manufactured Nanomaterials with the International Organization for Standardization to provide scientific advice for the safety use of nanomaterials that include the respective physicochemical characterization and the metrology. However, there is not an effective list of minimum parameters. The following characteristics should be a starting point to the characterization: particle size, shape and size distribution, aggregation and agglomeration state, crystal structure, specific surface area, porosity, chemical composition, surface chemistry, charge, photocatalytic activity, zeta potential, water solubility, dissolution rate/kinetics, and dustiness (McCall et al., 2013; Lin et al., 2014).

Concerning the chemical composition, nanomaterials can be classified as organic, inorganic, crystalline or amorphous particles and can be organized as single particles, aggregates, agglomerate powders or dispersed in a matrix which give rise to suspensions, emulsions, nanolayers, or films (Luther, 2004).

Regarding dimension, if a nanomaterial has three dimensions below 100 nm, it can be for example a particle, a quantum dot or hollow sphere. If it has two dimensions below 100 nm it can be a tube, fiber or wire and if it has one dimension below 100 nm it can be a film, a coating or a multilayer (Luther, 2004).

Different techniques are available for the analysis of these parameters. They can be grouped in different categories, involving counting, ensemble, separation and integral methods, among others (Linsinger et al., 2012; Contado, 2015).

#### Counting methods

Counting methods make possible the individualization of the different particles that compose a nanomaterial, the measurement of their different sizes and visualization of their morphology. The particles visualization is preferentially performed using microscopy methods, which include several variations of these techniques. Transmission Electron Microscopy (TEM), High-Resolution TEM, Scanning Electron Microscopy (SEM), cryo-SEM, Atomic Force Microscopy and Particle Tracking Analysis are just some of the examples. The main disadvantage of these methods is the operation under high-vacuum, although recently with the development of cryo-SEM sample dehydration has been prevented under high-vacuum conditions (Linsinger et al., 2012; Contado, 2015; Hodoroaba and Mielke, 2015).

#### Fractionation methods

These methods involve two steps of sample treatment: the separation of the particles into a monodisperse fraction, followed by the detection of each fraction. Field-Flow Fractionation (FFF), Analytical Centrifugation (AC) and Differential Electrical Mobility Analysis are some of the techniques that can be applied. The FFF techniques include different methods which separate the particles according to the force field applied. AC separates the particles through centrifugal sedimentation (Linsinger et al., 2012; Contado, 2015; Hodoroaba and Mielke, 2015).

#### Ensemble methods

Ensemble methods allow the report of intensity-weighted particle sizes. The variation of the measured signal over time give the size distribution of the particles extracted from a combined signal. Dynamic Light Scattering (DLS), Small-angle X-ray Scattering (SAXS) and X-ray Diffraction (XRD) are some of the examples. DLS and QELS are based on the Brownian motion of the sample. XRD is a good technique to obtain information about the chemical composition, crystal structure and physical properties (Linsinger et al., 2012; Contado, 2015; Hodoroaba and Mielke, 2015).

#### Integral methods

The integral methods only measure an integral property of the particle and they are mostly used to determine the specific surface area. Brunauer Emmet Teller is the principal method used and is based on the adsorption of an inert gas on the surface of the nanomaterial (Linsinger et al., 2012; Contado, 2015; Hodoroaba and Mielke, 2015).

Other relevant technique is the electrophoretic light scattering (ELS) used to determine zeta potential, which is a parameter related to the overall charge a particle acquires in a particular medium. ELS measures the electrophoretic mobility of particles in dispersion, based on the principle of electrophoresis (Linsinger et al., 2012).

The Table 1 shows some of principal methods for the characterization of the nanomaterials including the operational principle, physicochemical parameters analyzed and respective limitations.

**Table 1:** Some of the principal methods for the characterization of the nanomaterials, operation principle, physicochemical parameters analyzed, and respective limitations (Luther, 2004; Linsinger et al., 2012; Lin et al., 2014; Contado, 2015; Hodoroaba and Mielke, 2015).

**Method**	**Operation principle**	**Physicochemical parameters analyzed**	**Limitations**
Transmission Electron Microscopy	An electron beam interacts and passes through the sample and the scattered electrons are focused to create an image.	Particle size and size distribution; Shape; Agglomeration; Aggregation; Crystal structure.	Operation in high-vacuum; Only applied for solid samples; Time consuming and expensive; Complex sample preparation;
Scanning Electron Microscopy	An electron beam interacts with the sample but the beam pass over the surface and due to the secondary electrons ejected from the surface by inelastic scattering occurs the creation of the image.	Particle size and size distribution; Shape; Agglomeration; Aggregation; Crystal structure.	Operate in High-Vacuum; Time consuming and expensive; Solid and conductive materials; Complex sample preparation.
Atomic Force Microscopy	A scanning probe moves over the surface of the sample and detects the surface topography by the forces measured from the interaction between both surfaces.	Particle size and size distribution; Shape; Agglomeration; Aggregation; Surface properties.	Samples must adhere to a substrate or be dispersed on it; Time consuming.
Particle Tracking Analysis	The sample is placed in a dark background and then it is illuminated by an intense laser light. The scattered light and the movement of particles under Brownian motion is measured through a sensitive camera on the optical microscope.	Particle size and size distribution; Agglomeration; Aggregation.	The sample must be a suspension; Less sensitive if the particles distances are small.
Field-Flow Fractionation	The separation of the particles occurs according to the differences in their mobility induced by a laminar flow field and after an interaction with a second perpendicularly field force.	Particle size distribution.	Complex algorithm to extract size distribution; Particles in agglomerates or aggregates are not determined.
Differential Electrical Mobility Analysis	The particle samples pass through an electric field and according to their electrical mobility (their charge) separation occurs.	Particle size and size distribution.	Only aerosol samples; Samples need to be charged.
Dynamic Light Scattering	The hydrodynamic diameter is determined through the measurement of the fluctuations of the scattered light caused by the particles Brownian motion in the suspension by Stokes-Einstein equation.	Particle size and size distribution.	Only applied for suspensions; Bad resolution for polydisperse samples.
X-ray Diffraction	A X-ray beam passes through the sample and interacts with the repeated planes of atoms. Atoms organized in a crystalline structure will diffract the beam. Through Bragg's Law the distance between the planes of atoms is calculated.	Particle Size; Shape; Structure for crystalline materials	Only applied for crystalline materials.
Brunauer Emmet Teller	This technique is based on the physical adsorption of an inert gas (N_2_ or Ar) at the surface of the particles at low temperature. By the number of adsorbed molecules on the surface, the surface area is calculated.	Specific surface area; Porosity	Only applied for dry samples.

### Process control—understanding the critical manufacturing steps

Another challenge in the pharmaceutical development is the control of the manufacturing process by the identification of the critical parameters and technologies required to analyse them (Gaspar, 2010; Gaspar et al., 2014; Sainz et al., 2015).

New approaches have arisen from the pharmaceutical innovation and the concern about the quality and safety of new medicines by regulatory agencies (Gaspar, 2010; Gaspar et al., 2014; Sainz et al., 2015).

Quality-by-Design (QbD), supported by Process Analytical Technologies (PAT) is one of the pharmaceutical development approaches that were recognized for the systematic evaluation and control of nanomedicines (FDA, 2004; Gaspar, 2010; Gaspar et al., 2014; Sainz et al., 2015; European Medicines Agency, 2017).

Note that some of the physicochemical characteristics of nanomaterials can change during the manufacturing process, which compromises the quality and safety of the final nanomedicine. The basis of QbD relies on the identification of the Quality Attributes (QA), which refers to the chemical, physical or biological properties or another relevant characteristic of the nanomaterial. Some of them may be modified by the manufacturing and should be within a specific range for quality control purposes. In this situation, these characteristics are considered Critical Quality Attributes (CQA). The variability of the CQA can be caused by the critical material attributes and process parameters (Verma et al., 2009; Riley and Li, 2011; Bastogne, 2017; European Medicines Agency, 2017).

The quality should not be tested in nanomedicine, but built on it instead, by the understanding of the therapeutic purpose, pharmacological, pharmacokinetic, toxicological, chemical and physical properties of the medicine, process formulation, packaging, and the design of the manufacturing process. This new approach allows better focus on the relevant relationships between the characteristics, parameters of the formulation and process in order to develop effective processes to ensure the quality of the nanomedicines (FDA, 2014).

According to the FDA definition “PAT is a system for designing, analzsing, and controlling manufacturing through timely measurements (i.e., during processing) of critical quality and performance attributes of raw and in-process materials and processes, with the goal of ensuring final product quality” (FDA, 2014). The PAT tools analyse the critical quality and performance attributes. The main point of the PAT is to assure and enhance the understanding of the manufacturing concept (Verma et al., 2009; Riley and Li, 2011; FDA, 2014; Bastogne, 2017; European Medicines Agency, 2017).

### Biocompatibility and nanotoxicology

Biocompatibility is another essential property in the design of drug delivery systems. One very general and brief definition of a biocompatible surface is that it cannot trigger an undesired' response from the organism. Biocompatibility is alternatively defined as “the ability of a material to perform with an appropriate response in a specific application” (Williams, 2003; Keck and Müller, 2013).

Pre-clinical assessment of nanomaterials involve a thorough biocompatibility testing program, which typically comprises *in vivo* studies complemented by selected *in vitro* assays to prove safety. If the biocompatibility of nanomaterials cannot be warranted, potentially advantageous properties of nanosystems may raise toxicological concerns.

Regulatory agencies, pharmaceutical industry, government, and academia are making efforts to accomplish specific and appropriate guidelines for risk assessment of nanomaterials (Hussain et al., 2015).

In spite of efforts to harmonize the procedures for safety evaluation, nanoscale materials are still mostly treated as conventional chemicals, thus lacking clear specific guidelines for establishing regulations and appropriate standard protocols. However, several initiatives, including scientific opinions, guidelines and specific European regulations and OECD guidelines such as those for cosmetics, food contact materials, medical devices, FDA regulations, as well as European Commission scientific projects (NanoTEST project, www.nanotest-fp7.eu) specifically address nanomaterials safety (Juillerat-Jeanneret et al., 2015).

In this context, it is important to identify the properties, to understand the mechanisms by which nanomaterials interact with living systems and thus to understand exposure, hazards and their possible risks.

Note that the pharmacokinetics and distribution of nanoparticles in the body depends on their surface physicochemical characteristics, shape and size. For example, nanoparticles with 10 nm in size were preferentially found in blood, liver, spleen, kidney, testis, thymus, heart, lung, and brain, while larger particles are detected only in spleen, liver, and blood (De Jong et al., 2008; Adabi et al., 2017).

In turn, the surface of nanoparticles also impacts upon their distribution in these organs, since their combination with serum proteins available in systemic circulation, influencing their cellular uptake. It should be recalled that a biocompatible material generates no immune response. One of the cause for an immune response can rely on the adsorption pattern of body proteins. An assessment of the *in vivo* protein profile is therefore crucial to address these interactions and to establish biocompatibility (Keck et al., 2013).

Finally, the clearance of nanoparticles is also size and surface dependent. Small nanoparticles, bellow 20–30 nm, are rapidly cleared by renal excretion, while 200 nm or larger particles are more efficiently taken up by mononuclear phagocytic system (reticuloendothelial system) located in the liver, spleen, and bone marrow (Moghimi et al., 2001; Adabi et al., 2017).

Studies are required to address how nanomaterials penetrate cells and tissues, and the respective biodistribution, degradation, and excretion.

Due to all these issues, a new field in toxicology termed nanotoxicology has emerged, which aims at studying the nanomaterial effects deriving from their interaction with biological systems (Donaldson et al., 2004; Oberdörster, 2010; Fadeel, 2013).

#### Evaluation methods

The evaluation of possible toxic effects of the nanomaterials can be ascribed to the presence of well-known molecular responses in the cell. Nanomaterials are able to disrupt the balance of the redox systems and, consequently, lead to the production of reactive species of oxygen (ROS). ROS comprise hydroxyl radicals, superoxide anion and hydrogen peroxide. Under normal conditions, the cells produce these reactive species as a result of the metabolism. However, when exposed to nanomaterials the production of ROS increases. Cells have the capacity to defend itself through reduced glutathione, superoxide dismutase, glutathione peroxidase and catalase mechanisms. The superoxide dismutase converts superoxide anion into hydrogen peroxide and catalase, in contrast, converts it into water and molecular oxygen (Nel et al., 2006; Arora et al., 2012; Azhdarzadeh et al., 2015). Glutathione peroxidase uses glutathione to reduce some of the hydroperoxides. Under normal conditions, the glutathione is almost totally reduced. Nevertheless, an increase in ROS lead to the depletion of the glutathione and the capacity to neutralize the free radicals is decreased. The free radicals will induce oxidative stress and interact with the fatty acids in the membranes of the cell (Nel et al., 2006; Arora et al., 2012; Azhdarzadeh et al., 2015).

Consequently, the viability of the cell will be compromised by the disruption of cell membranes, inflammation responses caused by the upregulation of transcription factors like the nuclear factor kappa β, activator protein, extracellular signal regulated kinases c-Jun, N-terminal kinases and others. All these biological responses can result on cell apoptosis or necrosis. Distinct physiological outcomes are possible due to the different pathways for cell injury after the interaction between nanomaterials and cells and tissues (Nel et al., 2006; Arora et al., 2012; Azhdarzadeh et al., 2015).

Over the last years, the number of scientific publications regarding toxicological effects of nanomaterials have increased exponentially. However, there is a big concern about the results of the experiments, because they were not performed following standard and harmonized protocols. The nanomaterial characterization can be considered weak once there are not standard nanomaterials to use as reference and the doses used in the experiences sometimes cannot be applied in the biological system. Therefore, the results are not comparable. For a correct comparison, it is necessary to perform a precise and thorough physicochemical characterization to define risk assessment guidelines. This is the first step for the comparison between data from biological and toxicological experiments (Warheit, 2008; Fadeel et al., 2015; Costa and Fadeel, 2016).

Although nanomaterials may have an identical composition, slight differences e.g., in the surface charge, size, or shape could impact on their respective activity and, consequently, on their cellular fate and accumulation in the human body, leading to different biological responses (Sayes and Warheit, 2009).

Sayes and Warheit (2009) proposed a three phases model for a comprehensive characterization of nanomaterials. Accordingly, the primary phase is achieved in the native state of the nanomaterial, specifically, in its dry state. The secondary characterization is performed with the nanomaterials in the wet phase, e.g., as solution or suspension. The tertiary characterization includes i*n vitro* and *in vivo* interactions with biological systems. The tertiary characterization is the most difficult from the technical point of view, especially *in vivo*, because of all the ethical questions concerning the use of animals in experiments (Sayes and Warheit, 2009).

Traditional toxicology uses of animals to conduct tests. These types of experiments using nanomaterials can be considered impracticable and unethical. In addition, it is time-consuming, expensive and sometimes the end points achieved are not enough to correctly correlate with what happens in the biological systems of animals and the translation to the human body (Collins et al., 2017).

*In vitro* studies are the first assays used for the evaluation of cytotoxicity. This approach usually uses cell lines, primary cells from the tissues, and/or a mixture of different cells in a culture to assess the toxicity of the nanomaterials. Different *in vitro* cytotoxicity assays to the analysis of the cell viability, stress, and inflammatory responses are available. There are several cellular processes to determine the cell viability, which consequently results in different assays with distinct endpoints. The evaluation of mitochondrial activity, the lactate dehydrogenase release from the cytosol by tretazolium salts and the detection of the biological marker Caspase-3 are some of the examples that imposes experimental variability in this analysis. The stress response is another example which can be analyzed by probes in the evaluation of the inflammatory response via enzyme linked immunosorbent assay are used (Kroll et al., 2009).

As a first approach, *in vitro* assays can predict the interaction of the nanomaterials with the body. However, the human body possesses compensation mechanisms when exposed to toxics and a huge disadvantage of this model is not to considered them. Moreover, they are less time consuming, more cost-effective, simpler and provide an easier control of the experimental conditions (Kroll et al., 2009; Fadeel et al., 2013b).

Their main drawback is the difficulty to reproduce all the complex interactions in the human body between sub-cellular levels, cells, organs, tissues and membranes. They use specific cells to achieve specific endpoints. In addition, *in vitro* assays cannot predict the physiopathological response of the human body when exposed to nanomaterials (Kroll et al., 2009; Fadeel et al., 2013b).

Another issue regarding the use of this approach is the possibility of interaction between nanomaterials and the reagents of the assay. It is likely that the reagents used in the *in vitro* assays interfere with the nanomaterial properties. High adsorption capacity, optical and magnetic properties, catalytic activity, dissolution, and acidity or alkalinity of the nanomaterials are some of the examples of properties that may promote this interaction (Kroll et al., 2009).

Many questions have been raised by the regulators related to the lack of consistency of the data produced by cytotoxicity assays. New assays for a correct evaluation of the nanomaterial toxicity are, thus, needed. In this context, new approaches have arisen, such as the *in silico* nanotoxicology approach. *In silico* methods are the combination of toxicology with computational tools and bio-statistical methods for the evaluation and prediction of toxicity. By using computational tools is possible to analyse more nanomaterials, combine different endpoints and pathways of nanotoxicity, being less time-consuming and avoiding all the ethical questions (Warheit, 2008; Raunio, 2011).

Quantitative structure-activity relationship models (QSAR) were one the first applications of computational tools applied in toxicology. QSAR models are based on the hypothesis that the toxicity of nanomaterials and their cellular fate in the body can be predicted by their characteristics, and different biological reactions are the result of physicochemical characteristics, such as size, shape, zeta potential, or surface charge, etc., gathered as a set of descriptors. QSAR aims at identifying the physicochemical characteristics which lead to toxicity, so as to provide alterations to reduce toxicology. A mathematical model is created, which allows liking descriptors and the biological activity (Rusyn and Daston, 2010; Winkler et al., 2013; Oksel et al., 2015).

Currently, toxigenomics is a new area of nanotoxicology, which includes a combination between genomics and nanotoxicology to find alterations in the gene, protein and in the expressions of metabolites (Rusyn et al., 2012; Fadeel et al., 2013a).

#### Nanotoxicological classification system

Hitherto, different risk assessment approaches have been reported. One of them is the DF4nanoGrouping framework, which concerns a functionality driven scheme for grouping nanomaterials based on their intrinsic properties, system dependent properties and toxicological effects (Arts et al., 2014, 2016). Accordingly, nanomaterials are categorized in four groups, including possible subgroups. The four main groups encompass (1) soluble, (2) biopersistent high aspect ratio, (3) passive, that is, nanomaterials without obvious biological effects and (4) active nanomaterials, that is, those demonstrating surface-related specific toxic properties. The DF4nanoGrouping foresees a stepwise evaluation of nanomaterial properties and effects with increasing biological complexity. In case studies that includes carbonaceous nanomaterials, metal oxide, and metal sulfate nanomaterials, amorphous silica and organic pigments (all nanomaterials having primary particle sizes smaller than 100 nm), the usefulness of the DF4nanoGrouping for nanomaterial hazard assessment has already been established. It facilitates grouping and targeted testing of nanomaterials, also ensuring that enough data for the risk assessment of a nanomaterial are available, and fostering the use of non-animal methods (Landsiedel et al., 2017). More recently, DF4nanoGrouping developed three structure-activity relationship classification, decision tree, models by identifying structural features of nanomaterials mainly responsible for the surface activity (size, specific surface area, and the quantum-mechanical calculated property “lowest unoccupied molecular orbital”), based on a reduced number of descriptors: one for intrinsic oxidative potential, two for protein carbonylation, and three for no observed adverse effect concentration (Gajewicz et al., 2018)

**Figure 3:**
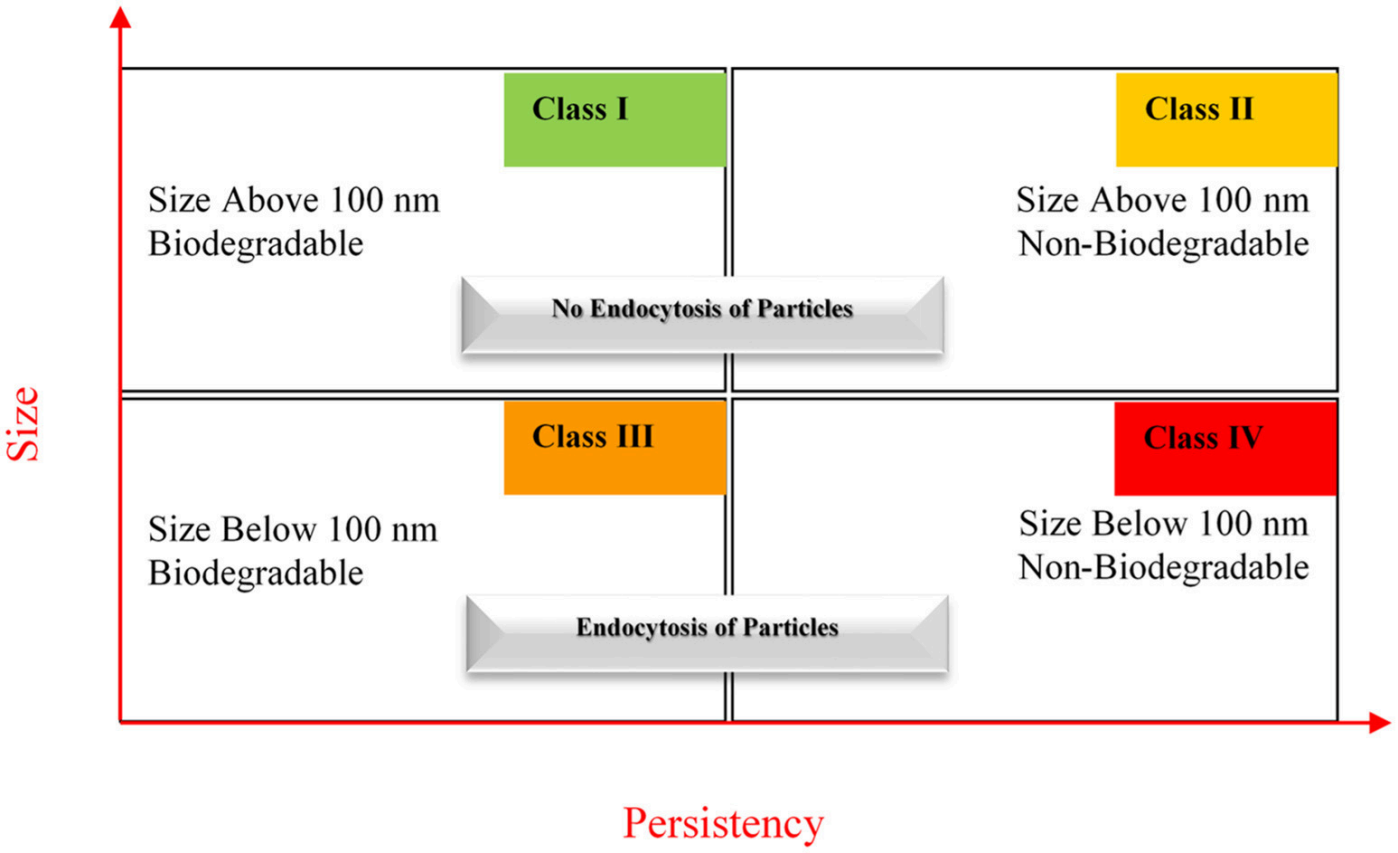
Nanotoxicological classification (reproduced with permission from Keck and Müller, 2013).

Keck and Müller also proposed a nanotoxicological classification system (NCS) (Figure 3) that ranks the nanomaterials into four classes according to the respective size and biodegradability (Müller et al., 2011; Keck and Müller, 2013).

Due to the size effects, this parameter is assumed as truly necessary, because when nanomaterials are getting smaller and smaller there is an increase in solubility, which is more evident in poorly soluble nanomaterials than in soluble ones. The adherence to the surface of membranes increases with the decrease of the size. Another important aspect related to size that must be considered is the phagocytosis by macrophages. Above 100 nm, nanomaterials can only be internalized by macrophages, a specific cell population, while nanomaterials below 100 nm can be internalized by any cell due to endocytosis. Thus, nanomaterials below 100 nm are associated to higher toxicity risks in comparison with nanomaterials above 100 nm (Müller et al., 2011; Keck and Müller, 2013).

In turn, biodegradability was considered a required parameter in almost all pharmaceutical formulations. The term biodegradability applies to the biodegradable nature of the nanomaterial in the human body. Biodegradable nanomaterials will be eliminated from the human body. Even if they cause some inflammation or irritation the immune system will return to the regular function after elimination. Conversely, non-biodegradable nanomaterials will stay forever in the body and change the normal function of the immune system (Müller et al., 2011; Keck and Müller, 2013).

There are two more factors that must be taken into account in addition to the NCS, namely the route of administration and the biocompatibility surface. When a particle is classified by the NCS, toxicity depends on the route of administration. For example, the same nanomaterials applied dermally or intravenously can pose different risks to the immune system.

In turn, a non-biocompatibility surface (NB) can activate the immune system by adsorption to proteins like opsonins, even if the particle belongs to the class I of the NCS (Figure 3). The biocompatibility (B) is dictated by the physicochemical surface properties, irrespective of the size and/or biodegradability. This can lead to further subdivision in eight classes from I-B, I-NB, to IV-B and IV-NB (Müller et al., 2011; Keck and Müller, 2013).

NCS is a simple guide to the evaluation of the risk of nanoparticles, but there are many other parameters playing a relevant role in nanotoxicity determination (Müller et al., 2011; Keck and Müller, 2013). Other suggestions encompass more general approaches, combining elements of toxicology, risk assessment modeling, and tools developed in the field of multicriteria decision analysis (Rycroft et al., 2018).

### Scale-up and reproducibility

A forthcoming challenge in the pharmaceutical development is the scale-up and reproducibility of the nanomedicines. A considerable number of nanomedicines fail these requirements and, consequently, they are not introduced on the pharmaceutical market (Agrahari and Hiremath, 2017).

The traditional manufacturing processes do not create three dimensional medicines in the nanometer scale. Nanomedicine manufacturing processes, as already mentioned above, compromise top-down and bottom-down approaches, which include multiple steps, like homogenization, sonication, milling, emulsification, and sometimes, the use of organic solvents and further evaporation. In a small-scale, it is easy to control and achieve the optimization of the formulation. However, at a large scale it becomes very challenging, because slight variations during the manufacturing process can originate critical changes in the physicochemical characteristics and compromise the quality and safety of the nanomedicines, or even the therapeutic outcomes. A detailed definition of the acceptable limits for the CQA is very important, and these parameters must be identified and analyzed at the small-scale, in order to understand how the manufacturing process can change them: this will help the implementation of the larger scale. Thus, a deep process of understanding the critical steps and the analytical tools established for the small-scale will be a greatly help for the introduction of the large scale (Desai, 2012; Kaur et al., 2014; Agrahari and Hiremath, 2017).

Another requirement for the introduction of medicines in the pharmaceutical market is the reproducibility of every batch produced. The reproducibility is achieved in terms of physicochemical characterization and therapeutic purpose. There are specific ranges for the variations between different batches. Slight changes in the manufacturing process can compromise the CQA and, therefore, they may not be within a specific range and create an inter-batch variation (Desai, 2012; Kaur et al., 2014; Agrahari and Hiremath, 2017).

## Regulatory challenges

### Nanomedicines in the pharmaceutical market

Over the last decades, nanomedicines have been successfully introduced in the clinical practice and the continuous development in pharmaceutical research is creating more sophisticated ones which are entering in clinic trials. In the European Union, the nanomedicine market is composed by nanoparticles, liposomes, nanocrystals, nanoemulsions, polymeric-protein conjugates, and nanocomplexes (Hafner et al., 2014). Table 2 shows some examples of commercially available nanomedicines in the EU (Hafner et al., 2014; Choi and Han, 2018).

**Table 2:** Examples of nanomedicines currently approved in the EU market (Hafner et al., 2014; Choi and Han, 2018; EMA)^1^.

**Nanomedicine class**	**Active substance/brand name**	**Pharmaceutical form**	**Therapeutic indications**
Nanoparticles	*Nab-paclitaxel* Abraxane®	Powder for suspension for infusion	Breast neoplasms Carcinoma non-small-cell lung Pancreatic neoplasma
	*Yttrium-90 radiolabelled ibritumomab tiuxetan* Zevalin®	Solution for infusion	Follicular Lymphoma
	*Glatiramer acetate* Copaxone®, Synthon®	Solution for injection	Multiple sclerosis
Liposomes	*Doxorubicin hydrochloride* Caelyx®	Concentrate for solution for infusion	Breast neoplasms Multiple myeloma Ovarian neoplasms Kaposi's sarcoma
	*Doxorubicin hydrochloride* Myocet®	Powder, dispersion and solvent for concentrate for dispersion for infusion	Metastatic breast cancer
	*Amphotericin B* AmBisome®	Powder for solution for infusion	Fungal infection
	*Daunorubicin* DaunoXome®	Concentrate for Solution for Infusion	Advanced HIV-related Kaposi's Sarcoma
	*Cytarabine* DepoCyte®	Suspension for injection	Lymphomatous meningitis
	*Mifamurtide* Mepact®	Powder for concentrate for dispersion for infusion	Osteosarcoma
	*Morphine* DepoDur®	Suspension for injection	Pain
Nanocomplex	*Verteporfin* Visudyne®	Powder for solution for infusion	Degenerative myopia, age-related macular degeneration
	*Ferumoxytol* Rienso®	Solution for infusion	Iron deficiency anemia in adult patients with chronic kidney disease
	*Ferric carboxymaltose* Ferinject®	Solution for injection/infusion	Iron deficiency
	*Iron(III) isomaltoside* Monofer®	Solution for injection/infusion.	Iron deficiency
	*Iron(III)-hydroxide dextran complex* Ferrosat®	solution for infusion or injection	Iron deficiency
Nanoemulsions	*Cyclosporine* Sandimmun Neoral®	Capsule, soft	Solid organ, bone marrow transplantation Endogenous uveitis Nephrotic syndrome Rheumatoid arthritis Psoriasis Atopic dermatitis
	*Pegaspargase* Oncaspar®	Solution for injection/infusion.	Acute lymphoblastic leukemia
Nanocrystals	*Paliperidone palmitate* Xeplion®	Prolonged release suspension for injection	Schizophrenia
	*Olanzapine pamoate* Zypadhera®	Powder and solvent for prolonged release suspension for injection	Schizophrenia
	*Aprepitant* Emend®	Capsule	Nausea and vomiting
	*Fenofibrate* Tricor® Lipanthyl® Lipidil®	Tablet	Hiperlipidemia
	*Sirolimus* Rapamune®	Tablet	Graft rejection Kidney transplantation
Polymer-protein conjugates	*Perginterferon ahpha-2b* PegIntron®	Powder and solvent for solution for injection	Chronic hepatitis C
	*Perginterferon ahpha-2a* Pegasys®	Solution for injection	Chronic hepatitis B and C
	*Pegfilgastrim* Neulasta®	Solution for injection	Leukopenia by chemotherapy
	*Methoxy polyethylene glycol-epoetin beta* Mircera®	Solution for injection in pre-filled syringe	Anemia associated with chronic kidney disease
	*Certolizumab pegol* Cimzia™	Solution for injection	Rheumatoid arthritis
	*Pegvisomant* Somavert®	Powder and solvent for solution for injection	Acromegaly

### Nanomedicines and nanosimilars

In the process of approval, nanomedicines were introduced under the traditional framework of the benefit/risk analysis. Another related challenge is the development of a framework for the evaluation of the follow-on nanomedicines at the time of reference medicine patent expiration (Ehmann et al., 2013; Tinkle et al., 2014).

Nanomedicine comprises both biological and non-biological medical products. The biological nanomedicines are obtained from biological sources, while non-biological are mentioned as non-biological complex drugs (NBCD), where the active principle consists of different synthetic structures (Tinkle et al., 2014; Hussaarts et al., 2017; Mühlebach, 2018).

In order to introduce a generic medicine in the pharmaceutical market, several parameters need to be demonstrated, as described elsewhere. For both biological and non-biological nanomedicines, a more complete analysis is needed, that goes beyond the plasma concentration measurement. A stepwise comparison of bioequivalence, safety, quality, and efficacy, in relation to the reference medicine, which leads to therapeutic equivalence and consequently interchangeability, is required (Astier et al., 2017).

For regulatory purposes, the biological nanomedicines are under the framework set by European Medicines Agency (EMA)^1^ This framework is a regulatory approach for the follow-on biological nanomedicines, which include recommendations for comparative quality, non-clinical and clinical studies (Mühlebach et al., 2015).

The regulatory approach for the follow-on NBCDs is still ongoing. The industry frequently asks for scientific advice and a case-by-case is analyzed by the EMA. Sometimes, the biological framework is the base for the regulation of the NBCDs, because they have some features in common: the structure cannot be fully characterized and the *in vivo* activity is dependent on the manufacturing process and, consequently, the comparability needs to establish throughout the life cycle, as happens to the biological nanomedicines. Moreover, for some NBCDs groups like liposomes, glatiramoids, and iron carbohydrate complexes, there are draft regulatory approaches, which help the regulatory bodies to create a final framework for the different NBCDs families (Schellekens et al., 2014).

EMA already released some reflection papers regarding nanomedicines with surface coating, intravenous liposomal, block copolymer micelle, and iron-based nano-colloidal nanomedicines (European Medicines Agency, 2011, 2013a,b,c). These papers are applied to both new nanomedicines and nanosimilars, in order to provide guidance to developers in the preparation of marketing authorization applications.The principles outlined in these documents address general issues regarding the complexity of the nanosystems and provide basic information for the pharmaceutical development, non-clinical and early clinical studies of block-copolymer micelle, “liposome-like,” and nanoparticle iron (NPI) medicinal products drug products created to affect pharmacokinetic, stability and distribution of incorporated or conjugated active substances *in vivo*. Important factors related to the exact nature of the particle characteristics, that can influence the kinetic parameters and consequently the toxicity, such as the physicochemical nature of the coating, the respective uniformity and stability (both in terms of attachment and susceptibility to degradation), the bio-distribution of the product and its intracellular fate are specifically detailed.

### Market access and pharmacoeconomics

After a nanomedicine obtains the marketing authorization, there is a long way up to the introduction of the nanomedicine in the clinical practice in all EU countries. This occurs because the pricing and reimbursement decisions for medicines are taken at an individual level in each member state of the EU (Sainz et al., 2015).

In order to provide patient access to medicines, the multidisciplinary process of Health Technology Assessment (HTA), is being developed. Through HTA, information about medicine safety, effectiveness and cost-effectiveness is generated so as support health and political decision-makers (Sainz et al., 2015).

Currently, pharmacoeconomics studies assume a crucial role previous to the commercialization of nanomedicines. They assess both the social and economic importance through the added therapeutic value, using indicators such as quality-adjusted life expectancy years and hospitalization (Sainz et al., 2015).

The EUnetHTA was created to harmonize and enhance the entry of new medicines in the clinical practice, so as to provide patients with novel medicines. The main goal of EUnetHTA is to develop decisive, appropriate and transparent information to help the HTAs in EU countries.

Currently, EUnetHTA is developing the Joint Action 3 until 2020 and the main aim is “to define and implement a sustainable model for the scientific and technical cooperation on Health Technology Assessment (HTA) in Europe.”

## Conclusion and prospects

The reformulation of pre-existing medicines or the development of new ones has been largely boosted by the increasing research in nanomedicine. Changes in toxicity, solubility and bioavailability profile are some of the modifications that nanotechnology introduces in medicines.

In the last decades, we have assisted to the translation of several applications of nanomedicine in the clinical practice, ranging from medical devices to nanopharmaceuticals. However, there is still a long way toward the complete regulation of nanomedicines, from the creation of harmonized definitions in all Europe to the development of protocols for the characterization, evaluation and process control of nanomedicines. A universally accepted definition for nanomedicines still does not exist, and may even not be feasible at all or useful. The medicinal products span a large range in terms of type and structure, and have been used in a multitude of indications for acute and chronic diseases. Also, ongoing research is rapidly leading to the emergence of more sophisticated nanostructured designs that requires careful understanding of pharmacokinetic and pharmacodynamic properties of nanomedicines, determined by the respective chemical composition and physicochemical properties, which thus poses additional challenges in regulatory terms.

EMA has recognized the importance of the establishment of recommendations for nanomedicines to guide their development and approval. In turn, the nanotechnology methods for the development of nanomedicines bring new challenges for the current regulatory framework used.

EMA have already created an expert group on nanomedicines, gathering members from academia and European regulatory network. The main goal of this group is to provide scientific information about nanomedicines in order to develop or review guidelines. The expert group also helps EMA in discussions with international partners about nanomedicines. For the developer an early advice provided from the regulators for the required data is highly recommended.

The equivalence of complex drug products is another topic that brings scientific and regulatory challenges. Evidence for sufficient similarity must be gathered using a careful stepwise, hopefully consensual, procedure. In the coming years, through all the innovation in science and technology, it is expected an increasingly higher number of medicines based on nanotechnology. For a common understanding among different stakeholders the development of guidelines for the development and evaluation of nanomedicines is mandatory, in order to approve new and innovative nanomedicines in the pharmaceutical market. This process must be also carried out along with interagency harmonization efforts, to support rational decisions pertaining to scientific and regulatory aspects, financing and market access.

## Author contributions

CV conceived the original idea and directed the work. SS took the lead in writing the manuscript. AP and JS helped supervise the manuscript. All authors provided critical feedback and helped shape the research, analysis and revision of the manuscript.

### Conflict of interest statement

The authors declare that the research was conducted in the absence of any commercial or financial relationships that could be construed as a potential conflict of interest.

## Footnotes

^1^Available online at: http://www.ema.europa.eu/ema/index.jsp?curl=pages/medicines/landing/epar_search.jsp&mid=WC0b01ac058001d124
